# 

**Published:** 1923-02

**Authors:** 


### ANNOUNCEMENTS

### THE AMERICAN ACADEMY OF PERIODONTOLOGY

The Tenth Annual Meeting of the American Academy of Periodontology will be held at the Hotel Statler, Cleveland, Ohio, September 7 and 8, 1923; just preceding the convention of the American Dental Association, in Cleveland, from September 11th to 14th inclusive.

The Sections of Orthodontia and Periodontia will conduct its meetings in the Ball Room of the Hotel Statler the week of September 11th, making it convenient for Academy members to attend these meetings.

The 1923 meetings of both the Academy and the American Dental Association promise to be the largest in the history of the organizations and it will be necessary for you to make reservations immediately with the Hotel Statler and state that you desire your reservations to continue through the week of the American Dental Association meeting. These reservations will be acknowledged by the Hotel, on special blanks provided for that purpose. This will assure you accommodations and prevent confusion on your arrival.

The Academy meeting will be called to order Friday, September 7th, at 10:00 A. M. in the Ball Room of the Hotel Statler. The Program Committee has already secured some of the best talent of the Medical and Dental professions for this meeting.

This being the Tenth meeting and held in Cleveland, the City where the Academy was organized, the Officers and Members of the Council are desirous of having every member attend this 1923 meeting.

You will receive further information as soon as the Program Committee and the Local Arrangements Committee has completed all the details for the meeting.

Write for accommodations to the Hotel Statler or one of the following hotels: Hotel Cleveland, Hotel Winton, Hollenden Hotel.

NOTICE THE DATE

The fifty-fourth annual meeting of the Kentucky State Dental Association will be held in Louisville, Kentucky, April 16-19, 1923.

Slogan—"Be a Booster." Headquarters—Seelbach Hotel.

*Officers*—E. C. Hume, President, 814 Francis Bldg., Louisville; N. B. Smith, Vice-President, Frankfort; R. L. Spratt, Treasurer, Mt. Sterling; W. M. Randall, Secretary, 1035 S. Second St., Louisville.

*Executive Committee*—R. L. Sprau, 968 Baxter Ave., Louisville; W. E. Goepper, Louisville; Chas. Pearcy, Louisville.

*Master of Exhibits and Editor of Program*—Geo. H. Means, Cherokee Apartments, Louisville.

*Board of Trustees*—E. C. Hume, President, Ex-officio; W. M. Randall, Secretary, Ex-officio; R. L. Spratt, Treasurer, Ex-officio; W. F. Walz, Bd. of Ex., Ex-officio; W. A. Bridwell, '25; J. E. Faulkner, '25; C. H. Tandy, '25; E. P. Marcilliat, '24; P. H. Williams, '24; Guy Clark, '24; Walter Matthews, '23; W. G. Best, '23.

*Dento-Legal Committee*—I. H. Harrington, Chairman, Louisville, Ky.; W. M. Randall, Louisville, Ky.; R. L. Spratt, Mt. Sterling, Ky.

*Clinic Committee*—J. H. Fullenwider, Chairman, Louisville, Ky.; A. S. Ingman, Louisville, Ky.; Joe Seldon, Louisville, Ky.; Chas. Mathis, Louisville, Ky.; J. B. Robarts, Louisville, Ky.; A. T. Wooten, Central City, Ky.; C. P. Mayhall, Harlan, Ky.

*Ethical Committee*—Oscar Flener, Chairman, Hopkinsville, Ky.; H. G. Hazelrig, Paintsville, Ky.; M. B. Guthrie, Lexington, Ky.

*Oral Hygiene Committee*—O. D. Wilson, Chairman, Owensboro, Ky.; J. F. Nevitt, Lexington, Ky.; G. H. Heyman, Louisville, Ky.

*Dental Research and Invention Committee*—O. B. Powell, Paducah, Ky.; Frank Hower, Louisville, Ky.; H. J. Patrick, Paint Lick, Ky.

*Auditing Committee*—R. P. Keene, Chairman, Owensboro, Ky.; C. J. Zimmer, Lexington, Ky.; H. L. Duncan, Dixon, Ky.

*Transportation Committee*—Joe Jones, Chairman, Dawson Springs, Ky.; O. B. Heaverin, Owensboro, Ky.; X. E. Sanders, Bowling Green, Ky.

*Press Committee*—Harry Kettig, Chairman, Louisville, Ky.; J. H. Gunkle, Newport, Ky.; W. T. Hays, Providence, Ky.; J. H. Feamster, Frankfort, Ky.

*Necrology Committee*—Power Wolfe, Chairman, Princeton, Ky.; C. A. Sanders, Perryville, Ky.; W. G. Best, Berea, Ky.

*Membership Committee*—E. P. Marcilliat, Chairman, Starks Bldg., Louisville, Ky.; All secretaries of component societies.

*Publication Committee*—W. M. Randall, Chairman, Ex-officio, Louisville, Ky.; W. W. Burgin, Campbellsville, Ky.; T. W. Lander, Eddyville, Ky.

*Entertainment Committee*—A. P. Williams, Chairman, Louisville, Ky.; H. Nichols, Louisville, Ky.; R. P. Thomas, Louisville, Ky.; A. T. Mock, Louisville, Ky.

*Science and Literature Committee*—Hugh McElrath, Chairman, Murray, Ky.; C. R. Ellis, Shelbyville, Ky.; S. G. Walton, Newport, Ky.

*Board of Censors*—E. B. Hardin, Chairman, Madisonville, Ky.; J. O. McCawley, Sturgis, Ky.; T. D. Kelly, Jr., Lexington, Ky.

*Legislative Committee*—E. D. Rose, Chairman, Bowling Green, Ky.; Others to be announced later.

The fifty-fourth annual meeting of the Kentucky State Dental Association which takes place April 16-19, 1923, is going to be a banner convention for this organization. All the committees, which comprise many of the prominent dentists of the state, are working hard to make the meeting interesting and attractive to the profession so that all who attend the clinics and meeting this year will be repaid for their visit to Louisville and can take home new enthusiasm and valuable, up-to-date information of the progress of their great calling. The exhibits will be particularly interesting and there will be exhibited the latest in specialties and fixtures besides the standard supplies with which the dentists are so familiar. The get-together spirit is going to be a feature of the meeting and it is earnestly desired by the executive heads of the various committees that all Kentucky dentists set aside April 16th to 19th for a trip to Louisville. Reservations at the hotel should be made now.

---

THE RESEARCH DIVISION OF THE DENTISTS' SUPPLY COMPANY

The development in technic in Denture Prosthesis, or, as it is commonly known, ''plate work,'' has been steady during the past fifteen years. Today, it is possible for any dentist of average technical ability to get results in this field which, though desired by the most skilled dentists in the past, had been all but impossible of realization, except in a few instances, due to lack of definite knowledge of the fundamental principles involved. Whatever technic you prefer, the same fundamentals must govern.

The Research Division of The Dentists' Supply Company, through co-operation of the same minds which made Trubyte Teeth possible, has co-related advanced ideas in Denture Prosthesis, coming from a great many sources, into a technic known as ''Professional Denture Service.'' This technic embodies all the fundamentals necessary to successful denture construction.

To meet the requirements of conditions affecting many general practices, modifications have recently been made in the technic. These modifications, such as muscle-trimmed plaster impressions, are taught in the courses given by the teachers of the Research Division in addition to the technic outlined in "Professional Denture Service," in the hope that better denture service may thereby be made possible under all conditions of practice.

The demand for postgraduate instruction in Denture Prosthesis is being met by many Dental Schools, and it is possible that in the near future, the Research Division will discontinue to a large degree its efforts in the field of postgraduate instruction, so that the opportunity herewith offered of getting instruction in your own vicinity may not be presented to you from this source again.

Among the important details taught by Dr. Turner, who will conduct a series of classes are:

1. Securing the correct central occlusion relation, or "taking the bite." This is the most important single step in denture prosthesis. It should be mastered by every practitioner. Many failures in denture work are due to lack of definite technic for this step.

2. Upper and lower impressions that have suction and that stay in place under masticating stress.

3. Arrangements of teeth in dentures for esthetic effect and to promote efficiency in mastication.

This course will be a Lecture Demonstration course, requiring five consecutive evening sessions to complete. Hours are from 8:00 to 10:00 P. M. In conducting this short course Dr. Turner will clinic at the chair, using a patient to demonstrate chair technic. He also will demonstrate the essential laboratory steps. The course is limited to twenty-five members, with a fee of $20.00 per man.

You can not afford to miss this course!

Get in touch with Dr. Turner. Come loaded with questions and a determination to leave his classroom better able to make good dentures.

If you go into it seriously, the investment you make may easily come back to you in increased practice from the influence wielded by your next better pleased denture patient.

We are arranging to have Dr. Turner conduct a course in Cincinnati, commencing March 19th.

---

### THE REHWINKLE DENTAL SOCIETY
(Component of Ohio State)

The Rehwinkle Dental Society will hold its annual mid-winter meeting in the Masonic Temple, Chillicothe, Ohio, on March 16-17, 1923. It is intended to have all the clinics on Friday, the papers on Saturday, with a few clinics early in the morning, thus giving the exhibitors sufficient time to pack and out before supper Saturday. On Friday evening there will be an entertainment and supper.

---

The annual meeting of the West Virginia State Dental Society will be held at Wheeling, W. Va., May 21-23, 1923 in the Market Auditorium Building.

A large attendance is expected and it is hoped to make this the best meeting ever held in the state. There is an excellent room for the exhibitors, which will be held both day and night, with every convenience. 110 V. A.C. for those who will need electricity.

The sessions of the society will be held in a separate room, so there will not be any conflict between meetings and exhibitors.

One evening will be given entirely to the exhibitors, no meeting at that time.

